# Assessment and validation of a suite of reverse transcription-quantitative PCR reference genes for analyses of density-dependent behavioural plasticity in the Australian plague locust

**DOI:** 10.1186/1471-2199-12-7

**Published:** 2011-02-16

**Authors:** Marie-Pierre Chapuis, Donya Tohidi-Esfahani, Tim Dodgson, Laurence Blondin, Fleur Ponton, Darron Cullen, Stephen J Simpson, Gregory A Sword

**Affiliations:** 1 School of Biological Sciences, University of Sydney, Sydney, NSW 2006, Australia; 2 Centre de coopération internationale en recherche agronomique pour le développement Acridologie, TA A-50/D, F-34398, Montpellier, France; 3 Centre de Biologie et de Gestion des Populations, INRA, Campus International de Baillarguet CS 30016, 34988 Montferrier/Lez, France; 4 Department of Entomology, Texas A&M University, College Station, TX 77842-2475 USA

## Abstract

**Background:**

The Australian plague locust, *Chortoicetes terminifera*, is among the most promising species to unravel the suites of genes underling the density-dependent shift from shy and cryptic solitarious behaviour to the highly active and aggregating gregarious behaviour that is characteristic of locusts. This is because it lacks many of the major phenotypic changes in colour and morphology that accompany phase change in other locust species. Reverse transcription-quantitative polymerase chain reaction (RT-qPCR) is the most sensitive method available for determining changes in gene expression. However, to accurately monitor the expression of target genes, it is essential to select an appropriate normalization strategy to control for non-specific variation between samples. Here we identify eight potential reference genes and examine their expression stability at different rearing density treatments in neural tissue of the Australian plague locust.

**Results:**

Taking advantage of the new orthologous DNA sequences available in locusts, we developed primers for genes encoding 18SrRNA, ribosomal protein L32 (RpL32), armadillo (Arm), actin 5C (Actin), succinate dehydrogenase (SDHa), glyceraldehyde-3P-dehydrogenase (GAPDH), elongation factor 1 alpha (EF1a) and annexin IX (AnnIX). The relative transcription levels of these eight genes were then analyzed in three treatment groups differing in rearing density (isolated, short- and long-term crowded), each made up of five pools of four neural tissue samples from 5^th ^instar nymphs. SDHa and GAPDH, which are both involved in metabolic pathways, were identified as the least stable in expression levels, challenging their usefulness in normalization. Based on calculations performed with the geNorm and NormFinder programs, the best combination of two genes for normalization of gene expression data following crowding in the Australian plague locust was EF1a and Arm. We applied their use to studying a target gene that encodes a Ca^2+ ^binding glycoprotein, *SPARC*, which was previously found to be up-regulated in brains of gregarious desert locusts, *Schistocerca gregaria*. Interestingly, expression of this gene did not vary with rearing density in the same way in brains of the two locust species. Unlike *S. gregaria*, there was no effect of any crowding treatment in the Australian plague locust.

**Conclusion:**

Arm and EF1a is the most stably expressed combination of two reference genes of the eight examined for reliable normalization of RT-qPCR assays studying density-dependent behavioural change in the Australian plague locust. Such normalization allowed us to show that *C. terminifera *crowding did not change the neuronal expression of the *SPARC *gene, a gregarious phase-specific gene identified in brains of the desert locust, *S. gregaria*. Such comparative results on density-dependent gene regulation provide insights into the evolution of gregarious behaviour and mass migration of locusts. The eight identified genes we evaluated are also candidates as normalization genes for use in experiments involving other Oedipodinae species, but the rank order of gene stability must necessarily be determined on a case-by-case basis.

## Background

Locusts are an excellent model organism for analyses of phenotypic plasticity in behaviour and other traits [1]. Plastic phenotypic responses to crowding are expressed to varying degrees among insects in the orders Coleoptera, Lepidoptera, Hemiptera, and Orthoptera [2]. The expression of phase polyphenism, in which individuals can undergo extreme density-dependent changes in behaviour, physiology, colour and morphology, is a defining feature of locusts (Orthoptera: Acrididae) [3]. Among this complex suite of traits, behaviour is the first to respond to changes in local population density and lies at the heart of swarm formation and migration [1]. Locusts reared under low population density conditions develop into the shy and cryptic solitarious phase, whereas rearing at high population density results in the highly active and aggregating gregarious phase. As local population size increases, patchy resource distributions in a habitat tend to concentrate solitarious phase locusts. Cues associated with contact among individuals on these resources mediate the process of phase change, referred to as gregarization, and cause initially solitarious phase locusts to become attracted rather than repelled by others [4]. A positive feedback loop is then established that can drive an initially solitarious population into the swarming gregarious phase [1].

Along with major advances in our understanding of locust behaviour and ecology, substantial progress has been made toward understanding the neurophysiological mechanisms underlying the process of behavioural phase change [3]. However, unravelling the molecular genetic basis of the shift from solitarious to gregarious behaviours remains the "final frontier" in locust research [3]. In recent years, insight into the putative functions of specific candidate genes involved in phase polyphenism has been obtained through gene expression profiling analyses under different density conditions [5-7]. Moreover, Rahman et al. [8] applied the differential display PCR method [9] for locust brain tissue, the findings of which were confirmed by semi-quantitative reverse transcription PCR. The differential display PCR method systematically detects changes in mRNA profiles without the need for any prior knowledge of genomic information of the study organism. Functional genomics resources have also been developed for key locust species that will further foster the exploration of non-hypothesis driven high-throughput gene expression data resulting from microarrays [10-12]. Therefore, it is reasonable to expect gene expression studies to become increasingly prominent in analyses of locust phase polyphenism. These techniques will likely be extended to other non-model taxa provided they bear sufficient genetic similarity.

Although at least 23 grasshopper species show elements of density-dependent phase polyphenism, our understanding of locust behaviour is primarily based on studies of the desert locust, *Schistocerca gregaria*, and to a lesser extent, the migratory locust, *Locusta migratoria *[3]. The Australian plague locust, *Chortoicetes terminifera*, is one of Australia's most significant agricultural pests and has recently emerged as a new model of considerable interest for studying the transition between the two behavioural phases. Despite the lack of striking changes in colour and morphology, which are seen so prominently in *L. migratoria *and *S. gregaria*, *C. terminifera *was recently shown to exhibit full density-dependent behavioural gregarization within days of crowding [13,14]. Therefore, the quantification of density-dependent transcriptional responses in *C. terminifera *might be particularly suited to identify the suite of genes underlying locust behaviour plasticity, as it may avoid confounding transcriptional changes associated with the expression of additional density-dependent traits seen notably in other locust species [13]. Furthermore, because the ability to change phase from solitarious to gregarious in response to increased population density has evolved multiple times within the grasshopper family Acrididae and resulted in a phylogenetically heterogeneous group of 'locusts' [15-17], comparison of differential gene transcription across *C. terminifera, L. migratoria *and *S. gregaria *may provide insights into the evolution of gregarious behaviour and mass migration [13].

Reverse transcription-quantitative Polymerase Chain Reaction (RT-qPCR) is the most reproducible and sensitive method available to measure mRNA transcription levels for individual genes, and is often used to confirm results from high-throughput systems like microarrays. However, the quality of results is directly related to data normalization that eliminates template heterogeneity due to variations in initial sample amount, mRNA recovery and integrity, and reverse transcription efficiency [18,19]. The most common normalization technique uses internal standards, mainly housekeeping genes, so called because their transcription occurs in all nucleated cell types since they are necessary for cell survival [20]. The expression of housekeeping genes is often presumed to be stable across experimental procedures and cell types. Despite this assumption, numerous studies have shown that such housekeeping genes can be differentially expressed, thereby compromising their use as stable internal standards (e.g. [21]).

To avoid bias from normalization, the stable expression of candidate reference genes should be validated under specific experimental conditions. The use of a suite of multiple stably-expressed reference genes is currently the gold standard [19,20,22]. To this aim, statistical approaches have been developed to determine the best-suited genes for normalization from a panel of candidate genes in a given set of biological samples [e.g. [23,24]]. Accordingly, studies of the transcriptional stability of reference genes are becoming more common. Yet, to our knowledge, the only insects for which suites of reference genes have been validated and published are those of a few model organisms, namely *Apis mellifera *[25,26], *Tribolium castaneaum *[27] and *Bombyx mori *[28], as well as those of *Bombus terrestris *and *lucorum *[29], *Liposcelis bostrychophila *[30], and *S. gregaria *[31].

The study of Van Hiel et al. [31] in the desert locust, *S. gregaria*, provides an initial set of reference genes to be evaluated for rearing density-controlled experiments in locusts. The authors validated seven reference genes for their stability during development in brains, which are of critical importance to the neuronal and neuro-endocrine processes involved in behaviour. However, the stable expression of these genes across the different rearing density treatments that must be imposed for analyses of density-dependent phase polyphenism in locusts has not yet been examined. In the present study, we used the *S. gregaria *mRNA sequences from Van Hiel et al. [31], supplemented with data on *L. migratoria *[11], which is a member of the same subfamily as *C. terminifera *(Oedipodinae), to identify partial sequences for 8 orthologs of putative housekeeping genes in *C. terminifera*. We evaluated the stability of these genes in neural tissue as candidates for normalization in RT-qPCR assays to study transcriptional-changes involved in the initiation and maintenance of density-dependent behavioural phase change in the Australian plague locust. We then applied our RT-qPCR normalization in studying the variation in expression across treatment groups of the gregarious phase-specific gene identified by Rahman et al. [8] in brains of *S. gregaria*. This gene which was found to be dominantly expressed in crowded gregarious phase locusts [8], shares high homology with the gene that encodes the Ca^2+ ^binding glycoprotein *SPARC *(Secreted Protein Acidic and Rich in Cysteine). It modulates cell adhesion, is essential to mesoderm development and affects mobility [32-34]. The final overall goal in this study was to develop an accurate and comprehensive method of RT-qPCR for use in studies of locust phase polyphenism that complies with MIQE recommendations for essential quality information [19].

## Results

### Sample purity and concentration

RNA integrity was confirmed using denaturing gel electrophoresis and visualisation of intact rRNA subunits of 28S and 18S. The rRNA bands from *C. terminifera *samples were ~1.8 and 2.3 kb, the former showing greater intensity. The 28S rRNA (4-5 kb) of most invertebrates dissociates into two subunits under denaturing conditions due to the breaking of a phosphodiester bond in the primary structure, referred to as the 'hidden break' by Ishikawa [35]. It is likely that the site of the hidden break in the 28S molecule is determined by the size of the 18S rRNA (1.8 kb) [35]. At least one of the 28S fragments resulting from the hidden break is expected to have the same or similar electrophoretic mobility to 18S rRNA. Our gel banding pattern suggests that the intense 1.8 kb band contains the 18S rRNA and a smaller fragment of the 28S rRNA while the larger fragment of the 28S molecule is 2.3 kb. Formaldehyde (denaturing) agarose gels of the 15 *C. terminifera *total RNA samples can be seen in Additional file 1.

RNA quantities were assessed with a Nanodrop ND-1000 spectrophotometer (Nanodrop Technologies) and ranged between 153 and 368 ng μL^-1 ^of total RNA. Interestingly, pooled brains and thoracic ganglia from long-term crowded samples yielded 40% more total RNA than those from either the isolated or 24 h-crowded groups (t-tests, *P *= 0.03). This could possibly result from larger brain sizes of 5^th ^instar nymphs reared under long-term crowded condition as recently shown by Ott and Rogers [36] in the desert locust. All samples were assumed adequately free from protein contamination and (organic) salts since they showed 260/280 and 260/230 nm ratios higher than 1.9 and 1.7 respectively. Additional file 2 provides further details about total RNA sample purity and concentration.

### Expression level and PCR efficiency

**Figure 1:**
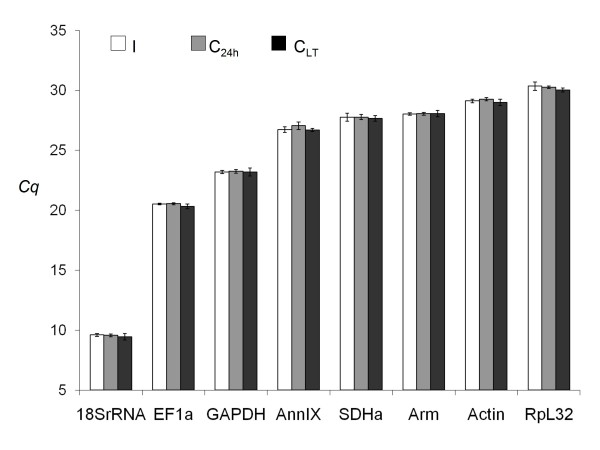
**Mean *Cq *values in RT-qPCR study**. Boxes and error bars represent the mean ± standard deviation over biological samples and technical replicates of *Cq *values computed from the second derivative method. Groups are isolated (I), 24 h-crowded (C_24 h_), and long-term crowded (C_LT_).

Six out of the 360 PCR reactions led to unexpected melt temperatures and were discarded. Figure 1 shows that all candidate reference genes except 18SrRNA were moderately abundant, with *Cq *values averaging from 20 for EF1a to 30 for RpL32. Expression of 18SrRNA was high in the tested samples, with *Cq *values averaging 9.5. The maximal standard deviation of *Cq *values for the three replicates was 0.42 over all biological samples and primer pairs (i.e. coefficient of variation of 1.4%). Table 1 shows the efficiency of RT-qPCR amplification for each primer pair. All but one slope of standard curves were -3.9 to -3.3, which translates in PCR efficiencies to 1.8- 2 fold increases per cycle. The exception was Actin with a slope of -4.5, i.e. PCR efficiency of 1.66 fold increase per cycle. The mean square errors of regression were between 0.01 and 0.08, below the recommended threshold of 0.2. The standard deviations of the estimated PCR efficiencies are reported in Table 1 from duplicated standard curves. All but one standard deviations were below 0.05 (i.e. coefficient of variation of 2.5%). Standard deviation reached 0.10 for SDHa (i.e. coefficient of variation of 5% ).

**Table 1:** Primer sequences, amplicon lengths and reaction efficiencies in RT-qPCR study.

Symbol	Primer sequence (5'-3')	*size* (bp)	*Tm* (°C)	*Primer*	*E* *(error)*	*SD(E)*
**18SrRNA**	F: CTGAGAAACGGCTACCACATC R: ACCAGACTTGCCCTCCAAT	171	84.9	0.25	1.91 (0.02)	0.02
**Arm**	F: ACTTCTTATGAGAGCATTCCAGGAT R: CCTTCAACAATTTCTTCCATGC	114	83.2	0.3	1.81 (0.03)	0.01
**EF1a**	F: AGCCCAGGAGATGGGTAAAG R: CTCTGTGGCCTGGAGCATC	155	81.4	0.3	1.99 (0.08)	0.04
**RpL32**	F: ACTGGAAGTCTTGATGATGCAG R: CTGAGCCCGTTCTACAATAGC	97	78.6	0.25	1.97 (0.05)	0.04
**GAPDH**	F: AATTGCCTGGCACCATTG R: CGCCACAACTTTCCAGATG	128	80.7	0.3	1.95 (0.06)	0.00
**Actin**	F: TTGTGTTGGATTCTGGTGATG R: GAAGCTGTAGCCCCTCTCAG	149	83.5	0.5	1.66 (0.01)	0.02
**SDHa**	F: CCACTGAAACTGATCCAAGAGAG R: TCCTGCTCCATTAACTAAGCAAC	98	76.2	0.3	1.90 (0.05)	0.10
**AnnIX**	F: GGAACTGATGAGGAAGCCATT R: TGGCCTGAAGTGTCTCCTTT	134	77.2	0.5	1.91 (0.04)	0.05

*size*: size of the cDNA amplicon; *Tm*: melt temperature of the PCR amplicon averaged over the 15 biological samples and 3 technical replicates; *Primer*: primer concentration in μM; *E*: in-run PCR reaction efficiency (fold increase per cycle) computed from the standard curve of a serial dilution included in the same plate as for samples S1 to S15 and using the formulae 10^-(1/-slope of the standard curve)^; *error*: the mean square error of the standard curve; *SD(E)*: standard deviation of the PCR efficiencies estimated from duplicated standard curves (different RT-qPCR runs and cDNA templates).

### Potential for gDNA amplification

Table 2 shows that primer pairs for Arm, Actin, SDHa, and AnnIX to be used in RT-qPCR runs failed to amplify genomic DNA. This is most likely because the primers flank an intron (e.g. see Actin in Additional file 3) which is too large for the 20s extension time in the PCR protocol (see Methods). An amplicon of 500 bp requires at least 20s extension time (LightCycler 480 SYBR Green I Master manual, version Feb 2008), so any target exceeding this length would fail to amplify.

**Table 2:** Details of PCR amplification of a genomic DNA template.

Symbol	size (bp)	*Tm* (°C)	*Cq* (cycles)
**18SrRNA**	~170	84.3	14.8
**Arm**	no amplification	no amplification	no amplification
**EF1a**^#^	light smear	3 peaks: 76.6; 79.8; 83.7	35.8
**RpL32**	~100	78.4	28.8
**GAPDH**^#^	light smear	79.8	34.9
**Actin**	~650	no amplification	no amplification
**SDHa**	no amplification	no amplification	no amplification
**AnnIX**	light smear	no amplification	no amplification

Genomic DNA amplicons were sized on an agarose gel following 30 cycles of PCR amplification using a Taq polymerase from Qiagen, a melting temperature of 58°C, and 50 ng of template. The melting temperatures (*Tm*) and *Cq *values were determined by quantitative PCR on a LightCycler 480 Instrument (Roche) as detailed in the main text and on 5 ng of genomic DNA. ^# ^means that primers span an exon-exon boundary. Alignments of complementary and genomic DNA sequences can be found for Actin, GAPDH, and EF1a in Additional file 3.

Any potential genomic DNA amplification of EF1a and GAPDH genes can be detected through the melt temperatures, which markedly differed from that of cDNA amplicons. In addition, gDNA *Cq *values for both genes were ≥ 10 cycles larger than the highest observed *Cq *value from our diluted cDNA samples (see Figure 1 for ranges of *Cq *values in our assay). The PCR inefficiency and different amplicon identity may result from partial and non-specific annealing although one primer spans exon-exon boundaries for both genes (see Additional file 3 for alignment data). In contrast, 18SrRNA and RpL32 primers amplified gDNA and cDNA fragments of similar size and melt temperature. gDNA *Cq *values were similar to those from our diluted cDNA samples, which makes these two genes susceptible to potential gDNA contamination in RT-qPCR. Nevertheless, none of the noRT samples were found to amplify these two genes in the present study. This is perhaps because the gDNA concentration was too low to result in a detectable product and thus all the samples were considered as non-contaminated.

### Expression stability

Table 3 shows that geNorm and NormFinder softwares ranked the eight candidate reference genes similarly. The four most stable genes were found to be Actin, EF1a, 18SrRNA, and Arm by both programs. Actin and EF1a was found to be the most stably expressed pair of genes in geNorm. Levels of pairwise variation between two sequential normalization factors were all below the cut-off value of 0.15 (see Additional file 4). In addition, the threshold for an unstable gene in geNorm is an average expression stability measure of 1.5, far above our maximal observed value of 0.212 for the least stable gene, SDHa. Thus, geNorm examination suggests that all eight candidate genes are stable enough to be used for normalization, and the addition of a third gene to the normalization is not imperative. The most stably expressed gene selected by NormFinder was Actin followed by EF1a then 18SrRNA. The best stability measure for a pairwise combination with Actin was provided by 18SrRNA (0.030) followed closely by EF1a (0.031). When comparing the long-term crowded and isolated groups only, Actin and EF1a remained the two reference genes of choice by using both programs (results not shown). EF1a and Actin also remained as the most stable genes when compared between 24 h crowded and isolated locusts (results not shown). Because the use of rRNA (as opposed to mRNA transcript) for normalization is debated due to its great abundance (see Discussion) and Actin primer pair efficiency was restrained (i.e. Efficiency = 1.66, see Table 1), we performed the geNorm and NormFinder analyses without 18SrRNA and Actin (see Table 3). The most stable combination of two genes selected was EF1a and Arm.

**Table 3:** Ranking and values for expression stability of potential reference genes in locust neural tissues.

NormFinder		geNorm	
*All 8 genes*	*Without 18SrRNA and Actin*	*All 8 genes*	*Without 18SrRNA and Actin*
Actin+18SrRNA (0.030)^*a*^	Arm+EF1a (0.038)^*a*^	Actin+EF1a (0.100)^*a*^	Arm+EF1a (0.163)^*a*^
Actin (0.036)	EF1a (0.051)	18SrRNA (0.114)	GAPDH (0.191)
EF1a (0.044)	AnnIX (0.075)	Arm (0.133)	AnnIX (0.208)
18SrRNA (0.054)	Arm (0.079)	AnnIX (0.163)	RpL32 (0.224)
Arm (0.069)	RpL32 (0.079)	RpL32 (0.180)	SDHa (0.237)
RpL32 (0.071)	SDHa (0.082)	GAPDH (0.195)	
AnnIX (0.073)	GAPDH (0.083)	SDHa (0.212)	
GAPDH (0.077)			
SDHa (0.081)			

Rankings are based on individual values and average values by stepwise exclusion for NormFinder and geNorm, respectively. ^a ^The best combination of two reference genes in terms of a paired stability value. In NormFinder, the best paired stability value is determined from the gene with the best individual stability value in combination with another gene. In geNorm, the best paired stability value is reached after stepwise exclusion of the least stable genes. Increasing values indicate decreasing stability.

### Gene expression of a target gene

In order to apply the selected set of reliable reference genes, we analyzed the relative expression of a target gene in the nymphal brains of *C. terminifera *under different crowding conditions. We designed forward and reverse primers (F-TCTGGAAATGGTGTGACTTGG and R-ATAAGTGGAGCACGGATTG) for RT-qPCR using primer sequences conserved within the *SPARC *orthologs for *L. migratoria *(LMC_004100 in LocustDB; [11]) and *S. gregaria *(AY751536; [8]). Size (85 bp) and singularity of PCR product was confirmed with gel electrophoresis and sequencing. PCR reaction efficiency with *SPARC *RT-qPCR primers at 0.5 μM was 1.80 (fold increase per cycle). No change in *SPARC *gene expression could be detected when comparing long-term or 24 hour crowded insects to solitary insects (*P *= 0.801 and 0.631). These results were obtained by using EF1a and Arm as reference genes and in-run PCR efficiency estimates. Using Actin and EF1a for normalization gave similar *p*-values and expression ratios.

## Discussion

Knowledge of mRNA transcription levels in response to crowding is central to understanding the molecular mechanisms and evolution of density-dependent phase polyphenism in locusts. Now that high-throughput systems are becoming more widely available, gene expression studies and in particular the use of RT-qPCR will become even more important for molecular genetics and evolutionary research in locusts. However, successful RT-qPCR experiments require multiple, carefully validated, reference genes to allow for reliable data normalization and determination of differentially expressed genes.

In this study, we identified eight appropriate reference genes that show invariant expression in neural tissue of the Australian plague locust, whether reared in a solitary state or exposed to different durations of crowding. All of the genes we evaluated were classified as stably expressed as each presented with geNorm stability indexes well below the cut-off of 1.5 [23]. In this study, we provide a comprehensive assessment of these reference genes for use in subsequent RT-qPCR assays.

Amongst the eight candidate housekeeping genes evaluated, Actin and EF1a were selected as the most stable pair by both geNorm and NormFinder. Although Vandesompele et al. [23] recommend the use of at least three reference genes for reliable normalization, the pairwise variation analysis in geNorm indicated that there is no need to include more than two genes. If we assume that stability is likely to be consistent across locust species, our results may serve as *a posteriori *validation of the use of Actin as a reference gene in previous studies of rearing density effect on locust gene expression patterns [5,6,8]. When comparing our results to the study of reference genes in brains of the desert locust, *S. gregaria *[31], we see that EF1a was also validated as stable with age in brains of fifth- instar nymphs and adults of the desert locust, *S. gregaria*. In contrast, Actin was excluded for normalization of transcriptional studies in brains of ageing adults. Interestingly, despite being amongst the most abundant proteins in all eukaryotic cells, with key roles in cell motility and cytoskeleton maintenance, Actins have been shown to vary in transcript levels with growth and ageing, at least in mammals [21]. The different ranking of the Actin gene across studies underlines the necessity for validation of the candidate reference genes prior to an experiment utilizing different treatments.

The 18S ribosomal subunit was classified as the third most stable gene in our study (Table 3). However, caution should be exercised when using 18SrRNA as a reference control. First, as ribosomal subunits are not polyadenylated they cannot be exploited in purified mRNA samples, i.e. in Reverse-Transcriptase reactions primed with oligo-dT. This was addressed in this experiment by using random hexamer primers only. Second, the high abundance of rRNA molecules compared with target mRNA transcripts, as revealed here by relatively low *Cq *values for 18SrRNA, makes it difficult to subtract the baseline value in fluorescence data analysis [23]. In the present study, accurate fluorescence treatment was only possible by a large dilution of the RT-qPCR template (i.e. 100-fold, equivalent of 0.25 ng total RNA). Furthermore, because 18SrRNA is so abundant, representing up to 80% of cellular RNA, it is expected that variations from sample-to-sample in initial sample amount, mRNA recovery and integrity, and reverse transcription efficiency, will be more difficult to detect. Furthermore, 18SrRNA is likely to experience RT-qPCR kinetics that are different to those of less abundantly expressed genes [37]. As a consequence of these factors, 18S rRNA can reduce the sensitivity to detect variation in relative levels of expression [38]. Because it is more likely to be expressed at a comparable order of magnitude as the investigated targets, we suggest that Arm will be a best suitable third normalizer in gene expression studies of locust neural tissues that address either the initiation or the maintenance of phase differences.

In our study, the two least stable genes were GAPDH and SDHa that code for the metabolic pathways enzymes, glycolysis and citrate cycle, respectively. Kristensen et al. [39] showed in *Drosophila melanogaster *that genes involved in metabolic processes are differentially expressed in response to heat stress. In particular, both glyceraldehyde 3 phosphate and succinate dehydrogenases were down-regulated at 36°C versus 25°C. This indicates that housekeeping metabolism is a dynamic process that can be regulated under different physiological states, thus challenging the usefulness of genes involved in metabolic processes in normalization. Crowded conditions themselves might be predicted to impose physiological stresses that can affect gene transcription. Accordingly, heat shock proteins, which are synthesized in response to stress in insects and act as molecular chaperones to mediate numerous cellular functions, have been shown to be up-regulated in heads of crowded gregarious phase migratory locusts [7].

RT-qPCR reactions showed a linear concentration-dilution relationship and were highly efficient, as every cycle of reaction amplification increased the amount of DNA template by 1.90-2 fold. The two exceptions were Armadillo and Actin primer pairs which respectively showed a moderate and low 1.81 and 1.66 fold increase per cycle. An exponential amplification of 2 (±10%), as retrieved for most of primer pairs, is needed when using the delta-delta-*Cq *quantification model as originally described by Livak and Schmittgen [40]. However, the most common efficiency-corrected methods currently in use for relative quantification [41] do not assume that all targets amplify with the same optimal PCR efficiency. Instead, efficiencies must be calculated prior to quantification, be reproducible and preferably be validated in each target gene RT-qPCR run. Our reaction efficiencies were shown to be reproducible, and therefore should be the same between standards and all of the samples in each of the future qPCR assays. However, we suggest to adopt the more conservative approach of avoiding low efficiency primer pairs (i.e. Actin), which would suggest EF1a and Arm as the best combination of two reference genes. These reference genes do not present any risk of misleading gDNA amplification.

We applied the use of EF1a and Arm for accurate normalization in gene expression studies of locust neural tissue by studying the target gene *SPARC *shown to be up-regulated in gregarious brains of desert locusts. We showed that 24 hours of crowding did not change the expression of *SPARC *in brains and thoracic ganglia of the Australian plague locust. Similarly, no expression change was detected with longer crowding. Similar results were found when using Actin as sole normalizer following Rahman et al. [8]. The discrepancy in *SPARC *expression between this and earlier studies may have arisen from other differences in experimental procedures; in particular, neural tissues were dissected from 4-5 day old adults in Rahman et al. [8], as opposed to 2-day old 5^th^-instar nymphs in our study, and *SPARC *expression is known to be developmentally regulated (reviewed in [42]). We might also expect *S. gregaria *and *C. terminifera *to differ in the density-dependence regulation of the glycoprotein, as their high density gregarious phase express different suites of behavioural [13,14] and morphological traits [43]. However, since the role of the *SPARC *protein with respect to density-dependent changes in behaviour or any other trait remain unknown, this alternative cannot be examined further as part of this study.

The reference genes we selected for gene expression normalization will provide a promising starting point for further studies of density-dependent transcriptional changes in the Australian plague locust. Now that appropriate primers had been identified for a range of potential reference genes, validation tests for other gene expression studies involving experimental treatments other than rearing density can also be performed in a relatively straightforward manner. Our findings should also facilitate the selection and use of suitable reference genes in the related migratory locust, *L. migratoria*, as the primers we used target the cDNA sequences of this species too. The possibility also remains that the primer sequences presented in this study are conserved across other members of the Oedipodinae which includes at least seven other species that show elements of density-dependent phase polyphenism [3].

## Conclusion

We propose that EF1a and Arm primer pairs evaluated here be used for accurate and reliable normalization of RT-qPCR results in gene expression studies in neural tissues of the Australian plague locust under different rearing density conditions.

## Methods

### Locust culture and experimental samples

A *C. terminifera *rearing colony was established in 2006 with approximately 25,000 locusts from north central Victoria (35°55'S, 144°25'E) and southwestern Western Australia (30°40'S, 116°15'E). The culture has since been maintained under crowded conditions with thousands of egg-pods establishing each generation. As a result, genetic variation among the individuals was recently shown to be substantial at neutral microsatellite loci, with 83% of the alleles and 97% of the gene diversity found in the field being retained in the lab [44]. In this study, we generated expression profiles of the reference gene transcripts from solitarious and gregarious phase locusts that had been reared long term under either isolated or crowded conditions, respectively, as well as solitarious phase individuals that had been subjected to short-term crowding for 24 hours as described below.

Crowd-reared juveniles were reared from hatching in 30 cm^3 ^steel mesh cages at densities of 200 - 300 locusts comparable to those of outbreaking field populations of *C. terminifera *[45,46]. First-instar nymphs were collected from the gregarious culture from different cages, each initiated with a minimum of 30 egg-pods, to establish isolated-reared cohorts in a separate constant temperature room with the same temperature (32°C) and photoperiod (14:10 L:D) as crowded-reared locusts (see Gray et al. [13] for further details on crowd- and isolated- rearing conditions). Behavioural phase change in the Australian plague locust does not accumulate across generations as known to occur in other locust species, but shifts completely within days in response to a change in immediate rearing density [13]. Therefore, we considered 5^th ^instar isolated nymphs to be fully solitarious, and their comparison to the long-term crowded locusts of similar age can potentially identify genes involved in the maintenance of behavioural differences between the phases. Given that behavioural phase change in *C. terminifera *begins to occur within just 6 hours of crowding [14], we also included a treatment group of solitarious 5th instar nymphs that had been removed from their individual rearing cages 24 h post-ecdysis and been crowded as described above for only 24 hours to examine the stability of putative reference genes between relatively short versus long-term gregarious phase insects. The three rearing density treatment groups are hereafter referred as to isolated, long-term crowded and 24 h-crowded.

All locusts from each of the three different treatments were removed from their rearing cages the day of their 5^th ^ecdysis and marked on the pronotum with correction fluid (Office Max) and coloured ink (Staedtler permanent Lumocolor) depending on their treatment. All locust neural tissues were dissected the second day of the 5^th ^larval stage. The brain, optic lobes and the three thoracic ganglia of each insect were dissected with RNAse-free instruments under a binocular microscope and immediately immersed in RNA*later *(Ambion). Tissue samples from each individual were kept in RNA*later *overnight at 4°C and then stored at -80°C until RNA extraction, which was performed on pooled tissues of 2 males and 2 females. Five replicates of such pooled samples were analyzed for each of the three groups (long-term crowded, isolated, and 24 h-crowded).

### Candidate reference genes and primer design

Details on the candidate reference genes examined are given in Table 4. Because no genome or full transcriptome sequence data is currently available for *C. terminifera*, a first set of primers was developed based on orthologous sequences from different insects, including *L. migratoria *(EST database LocustDB; [11]) and *S. gregaria *[31]. Accession numbers for orthologs and primer sequences are available in Additional file 5. For all study genes except 18SrRNA, PCR products varying in size from 305 to 937 bp were generated from *C. terminifera *cDNA templates. PCR products obtained from *C. terminifera *gDNA for EF1a, GAPDH, and Actin, were also sequenced to determine exon-intron boundaries (see Additional file 3). We were unable to amplify the other genes of interest, i.e. Arm, RpL32, SDHa, and AnnIX from gDNA, suggesting the presence of large introns. Products for EF1a, GAPDH, and Actin were cloned before sequencing to rule out the presence of multiple loci such has already been reported in some fish and crustaceans [47-49].

**Table 4:** Name, function and GenBank Accession numbers of potential reference genes for *C. terminifera.*

Symbol	Name	Molecular function	GenBank n°
**18SrRNA**	18SrRNA	Structural constituent of ribosome	na
**Arm**	Armadillo	Alpha-catenin binding; Cytoskeletal protein binding; Protein binding; Transcription (co)activator activity	HQ388818
**EF1a**	Elongation factor 1 alpha	Translation elongation factor activity; GTPase activity; GTP binding	HQ388819
**RpL32**	Ribosomal protein L32	Structural constituent of ribosome	HQ388820
**GAPDH**	Glyceraldehyde-3-phosphate dehydrogenase	Glyceraldehyde-3-phosphate dehydrogenase (phosphorylating) activity; NADH binding	HQ388821
**Actin**	Actin 5C	Structural constituent of cytoskeleton; ATP binding; Protein binding	HQ388822
**SDHa**	Succinate dehydrogenase	Succinate dehydrogenase activity; Succinate-coA ligase activity; FAD binding; electron carrier activity	HQ388823
**AnnIX**	Annexin IX	Actin binding; Calcium ion binding; Calcium-dependent phospholipid binding; Phospholipid binding	HQ388824

Name and Gene Ontology are that of *Drosophila melanogaster *orthologue (see accession no in Flybase column of the Additional file 5) following http://www.geneontology.org.

RT-qPCR primers were designed from *C. terminifera *cDNA sequences using Primer3Plus [50] with the amplified fragment length kept between 90 and 175 base pairs (see Table 1 for further details on primer pairs). Parameters for primer and dimer complementarities were set by default. In order to optimize PCR efficiency, we checked amplicons for secondary structures at the site of primer binding [51] with UNAFold using the DINAMelt web server [52,53]. Additionally, we avoided stable helix formations both within primer DNA sequences and internal to the amplicon. Primers were verified for specificity in silico using the *L. migratoria *transcriptome available at LocustDB [11] and MFEPrimer [54] at default settings. Size and singularity of PCR products was confirmed with gel electrophoresis.

### RNA isolation, reverse transcription and qPCR

For the preparation of each total RNA sample, pooled neural tissue (≤ 20 mg) was disrupted and homogenized in 1 ml Trizol (Ambion) using a Tissue Lyser (Qiagen) operating at 25 Hz for 40 s with 7 mm stainless steel beads. The homogenized samples were incubated for 15 minutes at room temperature and then centrifuged at 12,000 *g *for 10 minutes at 4°C. We decanted off 0.8 ml which was then vigorously mixed with 0.2 ml of chloroform. The RNA-containing upper aqueous phase was recovered after spinning at 12,000 *g *for 20 minutes at 4°C and 0.350 ml was transferred to a Qiagen genomic DNA eliminator spin column. The remaining steps were carried out according to Qiagen's RNeasy^® ^Plus Mini Kit instructions (i.e. from step 4 in the version from Oct 2005). Eluted total RNA samples were further subjected to a RNase-free DNAse I treatment (Ambion). A combination of on-column and in-solution DNase treatment was used to minimize genomic DNA contamination [51].

First strand cDNA was synthesized from 500 ng total RNA using the Superscript III VILO cDNA Synthesis kit (Invitrogen) with random hexamer primers according to the manufacturer's protocol. All samples were reverse transcribed together in a single run. We pooled together 5 μl of each cDNA sample to determine reaction efficiency of each of the qPCR assays by means of a standard curve consisting of ten-fold diluted samples (equivalent to a range of 0.005 to 5 ng total RNA). Remaining cDNA samples were diluted 20- (for SDHa only) or 100-fold with PCR-grade water (equivalent of 1.25 and 0.25 ng total RNA, respectively).

Reverse transcription-quantitative PCR was performed in a 384-well block using a LightCycler 480 Instrument (Roche Diagnostics). Every PCR assay contained a final volume of 5 μl, including 2.5 μl 2 × SYBR-Green I Master Mix (Roche Diagnostics), 1 μl diluted cDNA template, 0.25 to 0.5 μl (250 to 500 nM final concentration; see Table 1) of each primer, and nuclease free water. PCR was carried out with an initial 10 min hot start activation of the polymerase at 95°C followed by 45 cycles of 10 sec denaturation at 95°C, 10 sec annealing at 60°C, and 20 sec extension at 72°C. Melt curve analysis was performed after completion of the thermal PCR program, 55-90°C with increments rising by 0.5°C each step and a 5s hold at each degree. This ensured the resulting fluorescence of PCR products originated from the single PCR product of interest rather than primer dimers or non-specific PCR products. Samples were run in triplicate and standard curves were included in each PCR run. In addition, a no-template control (NTC) was included for each primer pair. We used a Biomek NXP Span-8 Laboratory Automation Workstation (Beckman Coulter) to limit technical errors in pipetting.

Prior to the quantitative PCR assay, PCR cycling was performed on control reactions without reverse transcriptase (noRTs) for all cDNA samples and primer pairs. Finally, a gDNA template was also run for each primer pair to determine their sensitivity to gDNA contamination. We used an intentionally large amount of gDNA template (5 ng), equal to that of the least diluted sample of the standard curve (in equivalent of total RNA).

### Statistical treatment

The fluorescence data were processed using LightCycler 480 software version 1.5.0 (Roche Diagnostics) with two different methods, namely the absolute quantification fit point and second derivative methods. Because it was highly accurate and reproducible, we hereafter present results from the second derivative method only (details on the fit point method and results can be found in Additional file 6). Quantitative cycles (*Cq*) are determined from the amplification curve's second derivative maximum, which corresponds to the point where the acceleration of the fluorescence signal is at its maximum [55]. This method assumes that the curve shape is more predictive of starting concentration than is the fluorescence level of the curve [56]. The background fluorescence was computed as the arithmetic mean of cycles 2 to 6 and subtracted from the fluorescence values. The mean, standard deviation, and coefficient of variation of the raw triplicate *Cq *values were determined. For each primer pair, biological samples whose coefficient of variation was greater than 1.5% were inspected; a replicate reaction was considered an outlier if it deviated more than one standard deviation from the mean and was excluded from analysis [57].

To identify the optimal normalization genes among our set of candidates, we used software programs, geNorm [23] and NormFinder [24]. Both programs rank the candidate reference genes according to their expression stability in a given sample set where an increase in the program-designated value correlates with a decrease in gene expression stability. geNorm measures the expression stability of a reference gene as the average pairwise variation for that gene with all other tested reference genes. The program geNorm also calculates the pairwise variation between two sequential normalization factors to determine the optimal number of reference genes required for normalization. A pairwise variation value above 0.15 indicates that the added gene has a significant effect and should preferably be included in the normalization. Contrary to geNorm, NormFinder determines the stability of a reference gene based on the experimental design, with top-ranked genes having the least variation accumulated within and between groups (i.e. long-term crowded, isolated, and 24 h-crowded in this study). The gene with the smallest quality value is then selected and its value combined with each of the other genes to produce a stability measure for the combination of two genes. Both tools require the transformation of *Cq *values to linear scale expression quantities. For each gene, mean *Cq *values of the 15 biological samples were converted into relative quantities by using the formula *E*^-(*Cq*[control] -*Cq*[sample])^, with *E *denoting the exponential amplification efficiency (*E *= 10^-(1/-slope of the standard curve)^) and the sample with the lowest mean *Cq *value used as a control [58]. We evaluated the reproducibility of PCR amplification efficiency for all pairs of primers by using constant reaction conditions but a different cDNA sample in a different run plate.

To estimate potential up or down regulation of the *SPARC *gene in response to our different rearing density treatments, we used the software REST 2009 [59] with 2,000 random pairings of untreated controls (i.e. isolation) and treatment samples (i.e. crowding) to calculate confidence intervals and estimate the statistical significance of calculated expression ratios. The software calculates an efficiency-corrected relative quantity for each randomization pair by using the formula *E*^-(^^*Cq*^^[control] -^^*Cq*^^[sample])^. The expression ratio is then normalized using the geometric average of the relative quantity values for the two most stably-expressed reference genes selected by geNorm and NormFinder.

## Authors' contributions

The study was conceived and coordinated by GAS, MPC and SJS. MPC, DTE and GAS designed the experimental protocol. TD reared the locusts with the help of MPC. DTE performed the dissections. MPC selected the reference genes and, designed and tested the primers used for gene sequencing and for RT-qPCR assays. DC participated in the design of primers used for sequencing. LB cloned and sequenced PCR products of Actin, EF1a, and GAPDH. MPC performed the RT-qPCRs and carried out the statistical analyses. FP gave valuable expertise in RT-qPCR analyses. MPC wrote the manuscript with help from GAS. DTE, FP and SJS made significant comments on the manuscript. All authors read and approved the final manuscript.

## Supplementary Material

Additional file 1 **Formaldehyde agarose gels of the 15 *C. terminifera *total RNA samples**. The sizes of the molecular weight markers are indicated in kilobases (kb). The gel shows two discrete and intense rRNA bands without a leading smear. S1 to S15 refer to the fifteen samples and Additional file 2 provides details on their purity and concentration. Click here for file

Additional file 2 **Concentration and purity of the 15 *C. terminifera *total RNA samples**. Click here for file

Additional file 3 **Aligned DNA sequences from genomic (CtA23) and total RNA (Ct3A) templates for GAPDH, Actin, and EF1a**. The primer sequences for RT-qPCR are also provided. Amongst the 10 cloned PCR products, sequence variants that differed by a single nucleotide change were considered to result from artificial substitutions due to mis-incorporation during the PCR process. Indeed, by considering an error rate for the *Pfu *polymerase of 2 × 10^-6 ^per bp per duplication [60-62], the maximum expected numbers of bp changes per independent PCR was 0.08 for 35 cycles of PCR amplification (i.e. assuming a length of 1151 bp, which is that of the Actin gDNA sequence). Based on this error rate, we should expect no more than one mutation due to PCR mis-incorporation among 10 cloned sequences. Click here for file

Additional file 4 **Determination of the optimal number of reference genes required for normalization using geNorm**. The program calculates the pairwise variation (V) between two sequential normalization factors (in x-axis). V2/3 indicates the variation in the normalization factor using two *versus *three genes. A large pairwise variation V indicates that the added gene has a significant effect and should preferably be included in the normalization. Vandesompele et al. [23] suggest that the cut-off value for such significance should be 0.15. Click here for file

Additional file 5 **Primer sequences and accession numbers of orthologs used for generation of amplicons**. LocustDB no are unigene sequences from *L. migratoria*. All *C. terminifera *cDNA sequences from reference genes are deposited in GenBank (see Table 4). Click here for file

Additional file 6 **Results using the fit point method for estimating quantitative cycles**. Click here for file
