# Supplementary material for: Assessment and validation of a suite of reverse transcription-quantitative PCR reference genes for analyses of density-dependent behavioural plasticity in the Australian plague locust
Source: BMC Mol Biol. 2011 Feb 16;12:7. doi: 10.1186/1471-2199-12-7 (PMC3048552; doi:10.1186/1471-2199-12-7)
Supplement: Additional file 1 — Formaldehyde agarose gels of the 15 C. terminifera total RNA samples. The sizes of the molecular weight markers are indicated in kilobases (kb). The gel shows two discrete and intense rRNA bands without a leading smear. S1 to S15 refer to the fifteen samples and Additional file 2 provides details on their purity and concentration. [file 1471-2199-12-7-S1.DOC]

**Additional file 1. Formaldehyde agarose gels of the 15 *C. terminifera*** **total RNA samples.**


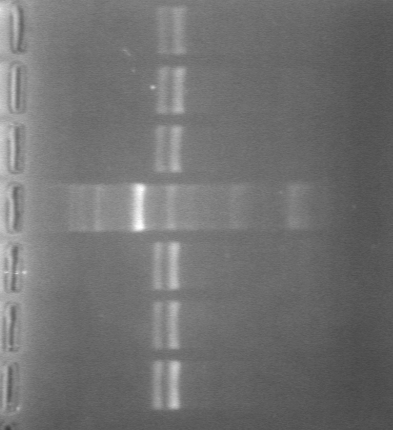

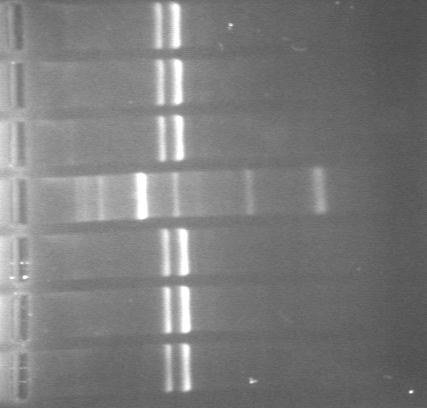

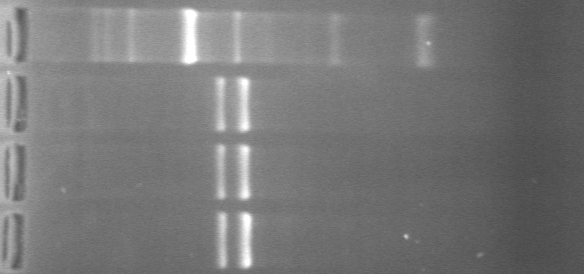


S1 S6 S11 S2 S7 S12 S3 S8 S13 S4 S9 S14 S5 S10 S15

3 kb

2 kb

1 kb

Legend: The sizes of the molecular weight markers are indicated in kilobases (kb). The gel shows two discrete and intense rRNA bands without a leading smear. S1 to S15 refer to the fifteen samples and Additional file 2 provides details on their purity and concentration.
