# Supplementary material for: Assessment and validation of a suite of reverse transcription-quantitative PCR reference genes for analyses of density-dependent behavioural plasticity in the Australian plague locust
Source: BMC Mol Biol. 2011 Feb 16;12:7. doi: 10.1186/1471-2199-12-7 (PMC3048552; doi:10.1186/1471-2199-12-7)
Supplement: Additional file 2 — Concentration and purity of the 15 C. terminifera total RNA samples. [file 1471-2199-12-7-S2.DOC]

**Additional file 2. Concentration and purity of the 15 *C. terminifera*** **total RNA samples.**

| **Sample** | **Treatment** | **Quantity [µg in 20 ul]** | **260/280** | **260/230** |
| --- | --- | --- | --- | --- |
| S1 | Six 2nd day 5th instar nymph-lifetime isolated insects | 3.8 | 2.08 | 2.22 |
| S2 | Six 2nd day 5th instar nymph-lifetime isolated insects | 3.8 | 2.07 | 2.18 |
| S3 | Six 2nd day 5th instar nymph-lifetime isolated insects | 4.8 | 2.08 | 2.26 |
| S4 | Six 2nd day 5th instar nymph-lifetime isolated insects | 5.9 | 2.07 | 2.13 |
| S5 | Six 2nd day 5th instar nymph-lifetime isolated insects | 4.1 | 2.06 | 2.33 |
| S6 | Six 2nd day 5th instar 24h crowded insects | 3.3 | 2.08 | 2.29 |
| S7 | Six 2nd day 5th instar 24h crowded insects | 4.5 | 2.05 | 2.28 |
| S8 | Six 2nd day 5th instar 24h crowded insects | 6.3 | 2.06 | 2.22 |
| S9 | Six 2nd day 5th instar 24h crowded insects | 3.7 | 2.05 | 1.96 |
| S10 | Six 2nd day 5th instar 24h crowded insects | 3.1 | 2.05 | 2.01 |
| S11 | Six 2nd day 5th instar long-term crowded insects | 5.0 | 2.06 | 2.19 |
| S12 | Six 2nd day 5th instar long-term crowded insects | 7.4 | 2.05 | 1.69 |
| S13 | Six 2nd day 5th instar long-term crowded insects | 5.5 | 2.07 | 2.21 |
| S14 | Six 2nd day 5th instar long-term crowded insects | 7.3 | 2.04 | 1.78 |
| S15 | Six 2nd day 5th instar long-term crowded insects | 5.5 | 2.06 | 2.13 |
