# Supplementary material for: Assessment and validation of a suite of reverse transcription-quantitative PCR reference genes for analyses of density-dependent behavioural plasticity in the Australian plague locust
Source: BMC Mol Biol. 2011 Feb 16;12:7. doi: 10.1186/1471-2199-12-7 (PMC3048552; doi:10.1186/1471-2199-12-7)
Supplement: Additional file 3 — Aligned DNA sequences from genomic (CtA23) and total RNA (Ct3A) templates for GAPDH, Actin, and EF1a. The primer sequences for RT-qPCR are also provided. Amongst the 10 cloned PCR products, sequence variants that differed by a single nucleotide change were considered to result from artificial substitutions due to mis-incorporation during the PCR process. Indeed, by considering an error rate for the Pfu polymerase of 2 × 10^-6^ per bp per duplication [60-62], the maximum expected numbers of bp changes per independent PCR was 0.08 for 35 cycles of PCR amplification (i.e. assuming a length of 1151 bp, which is that of the Actin gDNA sequence). Based on this error rate, we should expect no more than one mutation due to PCR mis-incorporation among 10 cloned sequences. [file 1471-2199-12-7-S3.DOC]

**Additional file 3. Aligned DNA sequences from genomic (CtA23) and total RNA (Ct3A) templates for GAPDH, Actin, and EF1a.**

Legend: The primer sequences for RT-qPCR are also provided. Amongst the 10 cloned PCR products, sequence variants that differed by a single nucleotide change were considered to result from artificial substitutions due to mis-incorporation during the PCR process. Indeed, by considering an error rate for the *Pfu* polymerase of 2 × 10^−6^ per bp per duplication [60-62], the maximum expected numbers of bp changes per independent PCR was 0.08 for 35 cycles of PCR amplification (i.e. assuming a length of 1151 bp, which is that of the Actin gDNA sequence). Based on this error rate, we should expect no more than one mutation due to PCR mis-incorporation among 10 cloned sequences.

**GAPDH**

**Actin**

**EF1a**
