# Supplementary material for: Assessment and validation of a suite of reverse transcription-quantitative PCR reference genes for analyses of density-dependent behavioural plasticity in the Australian plague locust
Source: BMC Mol Biol. 2011 Feb 16;12:7. doi: 10.1186/1471-2199-12-7 (PMC3048552; doi:10.1186/1471-2199-12-7)
Supplement: Additional file 4 — Determination of the optimal number of reference genes required for normalization using geNorm. The program calculates the pairwise variation (V) between two sequential normalization factors (in x-axis). V2/3 indicates the variation in the normalization factor using two versus three genes. A large pairwise variation V indicates that the added gene has a significant effect and should preferably be included in the normalization. Vandesompele et al. [23] suggest that the cut-off value for such significance should be 0.15. [file 1471-2199-12-7-S4.PPT]

## Slide 1
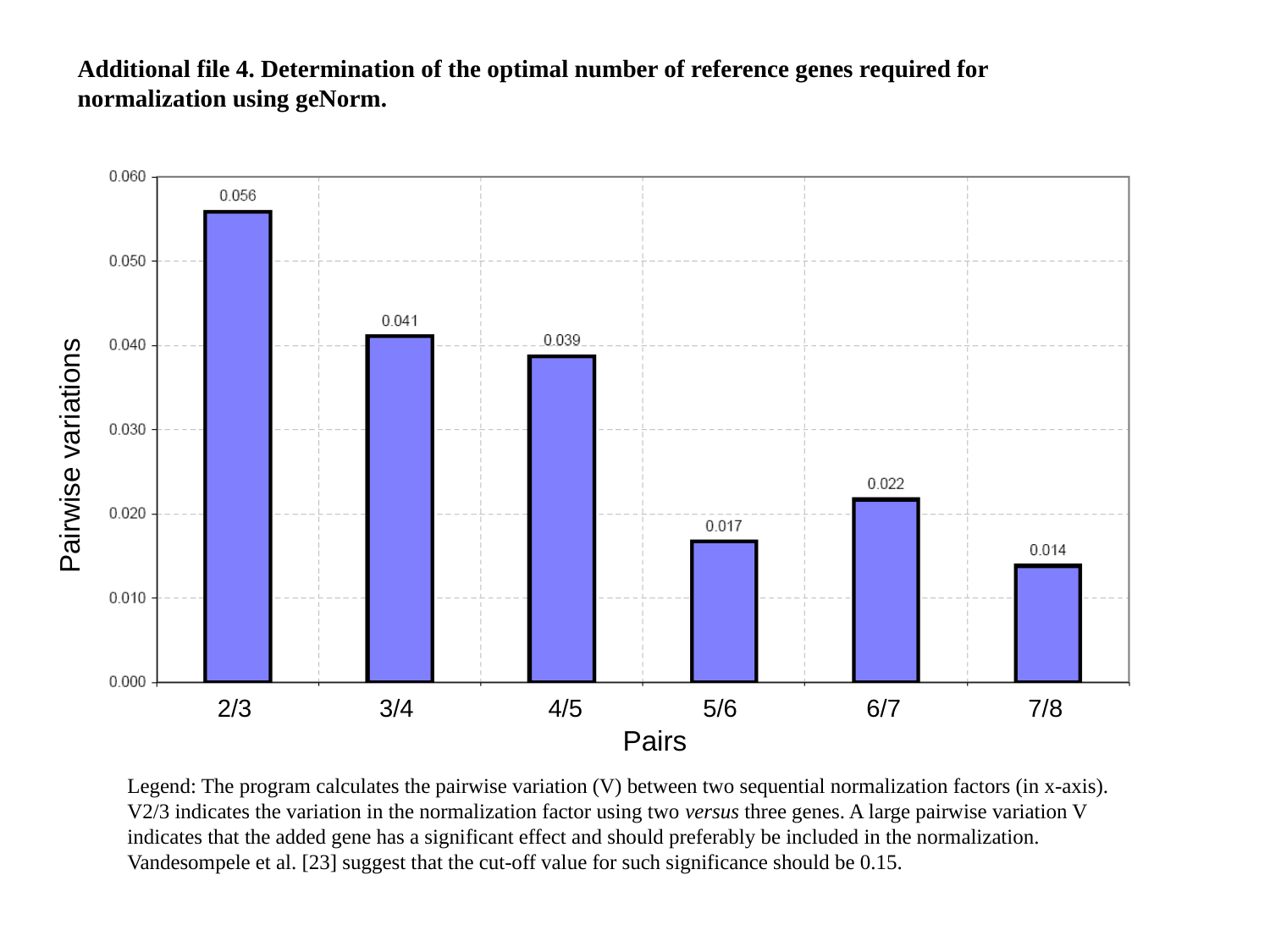

Additional file 4. Determination of the optimal number of reference genes required for normalization using geNorm.
Pairwise variations
2/3	 3/4	 4/5	 5/6	 6/7	 7/8
Pairs
Legend: The program calculates the pairwise variation (V) between two sequential normalization factors (in x-axis). V2/3 indicates the variation in the normalization factor using two versus three genes. A large pairwise variation V indicates that the added gene has a significant effect and should preferably be included in the normalization. Vandesompele et al. [23] suggest that the cut-off value for such significance should be 0.15.
