# Supplementary material for: Assessment and validation of a suite of reverse transcription-quantitative PCR reference genes for analyses of density-dependent behavioural plasticity in the Australian plague locust
Source: BMC Mol Biol. 2011 Feb 16;12:7. doi: 10.1186/1471-2199-12-7 (PMC3048552; doi:10.1186/1471-2199-12-7)
Supplement: Additional file 5 — Primer sequences and accession numbers of orthologs used for generation of amplicons. LocustDB no are unigene sequences from L. migratoria. All C. terminifera cDNA sequences from reference genes are deposited in GenBank (see Table 4). [file 1471-2199-12-7-S5.DOC]

**Additional file 5. Primer sequences and accession numbers of orthologs used for generation of amplicons.**

| **Symbol** |  | **Primer sequence (5’-3’)** | | | | | |  | **Orthologs** |  |
| --- | --- | --- | --- | --- | --- | --- | --- | --- | --- | --- |
|  |  |  | | | | | | LocustDB  accession no | *S. gregaria*  ID in Van Hiel et al. 2009 | Flybase  accession no |
|  |  | |  |  |  |  |  | | | |
| **Arm** |  | F: ACTTCTTATGAGAGCATTCCAGGAT  R: GCTCCTTCTTTGTCTGCTGCT | | | | | | LMC_002475 | na | CG11579 |
| **EF1a** |  | F: CGTCGTCATTGGTCACGTAG  R: TCAATAGACCATCCCTTGAACC | | | | | | LMC_004542 | LC.303.C1.Contig382 | CG8280 |
| **RpL32** |  | F: GGCGATTCGACCAGTGTATAG  R: GCATTGGTCACTCTGATGGATA | | | | | | LMC_003828 | LC.3836.C1.Contig3963 | CG7939 |
| **GAPDH** |  | F: TGTATGTTGTTGGTGTAAATTTGG  R: ACTGAAACATTTGGCACTGG | | | | | | LMC_004387 | LC.135.C1.Contig192 | CG8893 |
| **Actin** |  | F: TTGTGTTGGATTCTGGTGATG  R: CTGAAATGGTCTGGCATGATA | | | | | | LMC_004540 | LC.47.C2.Contig66 | CG4027 |
| **SDHa** |  | F: TACGTGCGTATTCCTTGCTG  R: CTCTCTCACACCACCTCCAA | | | | | | LMS_007440 | na | CG10622 |
| **AnnIX** |  | F: AGCGGTTGGAAATTGCTG  R: GCAACCAATCCAGAAGCAC | | | | | | LMC_004479 | na | CG5730 |
|  |  |  | | | | | |  |  |  |

Legend: LocustDB no are unigene sequences from *L. migratoria*. All *C. terminifera* cDNA sequences from reference genes are deposited in GenBank (see Table 4).
