# Supplementary material for: Assessment and validation of a suite of reverse transcription-quantitative PCR reference genes for analyses of density-dependent behavioural plasticity in the Australian plague locust
Source: BMC Mol Biol. 2011 Feb 16;12:7. doi: 10.1186/1471-2199-12-7 (PMC3048552; doi:10.1186/1471-2199-12-7)
Supplement: Additional file 6 — Results using the fit point method for estimating quantitative cycles. [file 1471-2199-12-7-S6.DOCX]

**Additional file 6: Results using the fit point method for estimating quantitative cycles.**

Quantitative cycles (*Cq*) were analyzed with the fit point method in which threshold crossing is determined at constant fluorescence level by fitting a line to two automated points in the log-linear phase of amplification [1]. This method assumes proportionality between initial template concentration and later cycle fluorescence level, as well as identical amplification efficiency in all samples [2]. The background fluorescence was computed as the arithmetic mean of cycles 2 to 6 and subtracted from the fluorescence values, as for the second derivative method used in the main text. In agreement with Luu-The et al. [3], *Cq* values were substantially lower in the fit point method than in the second derivative method (from 1 cycle for 18SrRNA to 6.5 cycles for Actin).We also observed a higher dispersion than with the second derivative method. For the fit point method, 23 exclusions of one replicate reaction were necessary to result in a coefficient of variation <1.5% for all 15 samples by 8 primer pairs. In contrast, for the second derivative method, the maximal standard deviation of *Cq* values for the three replicates was 0.42 over all biological samples and primer pairs (i.e. coefficient of variation of 1.4%) indicating a highly accurate and reproducible method. The table below shows that there were no significant differences in mean and dispersion measures between the second derivative and fit point methods in the efficiency of RT-qPCR amplification for each primer pair.

1. Rasmussen R: **Quantification on the LightCycler. In: Meuer, S., Wittwer, C., Nakagawara, K. eds. Rapid Cycle Real-time PCR, Methods and Applications SPringer Press, Heidelberg. pages 21-34.** 2001.

2. Durtschi JD, Stevenson J, Hymas W, Voelkerding KV: **Evaluation of quantification methods for real-time PCR minor groove binding hybridization probe assays**. *Analytical Biochemistry* 2007, **361**(1):55-64.

3. Luu-The V, Paquet N, Calvo E, Cumps J: **Improved real-time RT-PCR method for high-throughput measurements using second derivative calculation and double correction.** *Biotechniques* 2005, **38**(2):287-293.

# **Table. Reaction efficiencies in RT-qPCR study** **using either second derivative** **or fit point methods.** E: in-run PCR reaction efficiency (fold increase per cycle) computed from the standard curve of a serial dilution included in the same plate as for samples S1 to S15 and using the formulae 10^-(1/-slope of the standard curve)^; SD: second derivative method; FP: fit point method; error: the mean square error of the standard curve.

| **Symbol** | ***E_SD_***  ***(error)*** | ***E_FP_***  ***(error)*** |
| --- | --- | --- |
|  |  |  |
| **18SrRNA** | 1.91  (0.02) | 1.94  (0.05) |
| **Arm** | 1.81  (0.03) | 1.86  (0.03) |
| **EF1a** | 1.99  (0.08) | 1.96  (0.09) |
| **RpL32** | 1.97  (0.05) | 1.94  (0.13) |
| **GAPDH** | 1.95  (0.06) | 1.97  (0.08) |
| **Actin** | 1.66  (0.01) | 1.63  (0.05) |
| **SDHa** | 1.90  (0.05) | 2.23  (0.08) |
| **AnnIX** | 1.91  (0.04) | 1.93  (0.03) |
|  |  |  |
